# Gastropericardial fistula as a late complication of Roux-en-Y procedure

**DOI:** 10.1093/jscr/rjae491

**Published:** 2024-08-08

**Authors:** Eric M Bing, Amanda Skogsburg, Douglas Hayes

**Affiliations:** Internal Medicine, Skagit Regional Health, Graduate Medical Education, Mount Vernon 98274, United States; Medical Student, Skagit Regional Health, Graduate Medical Education, Mount Vernon 98274, United States; Internal Medicine Core Faculty, Skagit Regional Health, Graduate Medical Education, Mount Vernon 98274, United States

**Keywords:** gastric pericardial fistula, Roux-en-Y, gastric ulcer, pneumopericardium

## Abstract

Pneumopericardium secondary to gastro-pericardial fistula is a rare complication associated with various surgical procedures and conditions, notably Roux-en-Y gastric bypass. This condition poses a risk of cardiac tamponade and can be fatal if not promptly diagnosed and managed. We present a case of a 62-year-old female with a history of gastric bypass who presented with nonspecific symptoms and was eventually diagnosed with pneumopericardium secondary to gastro-pericardial fistula. Despite efforts for timely intervention, including transfer to a facility with cardiothoracic surgery availability, the patient’s unstable condition precluded surgical intervention, leading to her eventual demise. A literature review reveals that the average time from Roux-en-Y gastric bypass surgery to presentation is nine years. The elusive nature of the presentation underscores the importance of a comprehensive clinical history in identifying this condition early. Awareness of gastro-pericardial fistula as a potential late complication of gastric bypass is crucial for timely diagnosis and intervention to improve patient outcomes.

## Introduction

Pneumopericardium secondary to a gastro-pericardial fistula is a rare, often late-presenting complication of Roux-en-Y gastric bypass. It can also be seen after esophagectomy and fundoplication and can be associated with tumors, spontaneous peptic ulcers, and trauma [1]. An emergent concern from this is cardiac tamponade causing hypotension and respiratory distress and can ultimately be fatal. If found promptly, early intervention, typically surgery, can result in good outcomes. In this case, we observe a presentation and progression of gastro-pericardial fistula resulting in pneumopericardium as a late complication of a Roux-en-Y gastric bypass.

## Narrative

A 62-year-old female with a medical history of Roux-en-Y gastric bypass at age 47, anemia, arrhythmia, congestive heart failure, and hypertension presented to the emergency department with generalized weakness that she attributed to decreased oral intake. She also reported dyspnea on exertion, daily nausea and vomiting, and increasing lower extremity edema. Initial evaluation revealed leukocytosis, lateral ST-segment elevation, acute kidney injury, lung consolidation, and profound hypotension. Physiology presentation was similar to cardiac tamponade. The patient was admitted due to concerns of septic shock, necessitating vasopressors for stabilization.

**Figure 1:**
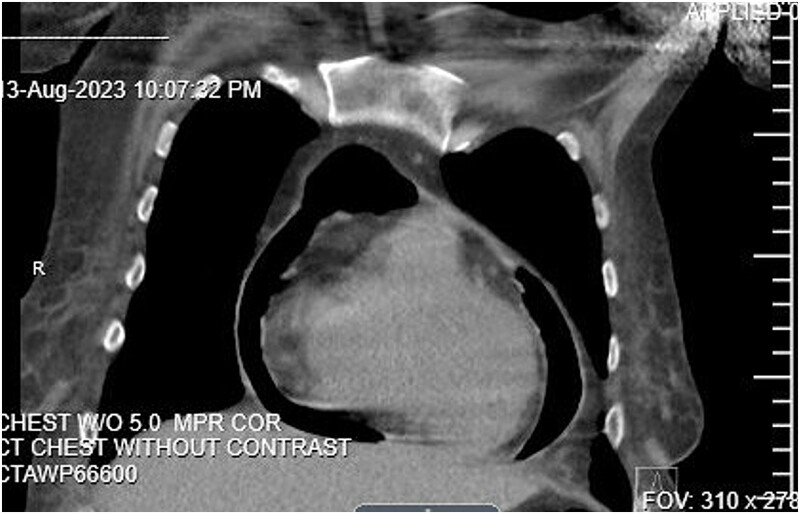
CT Abdomen pelvis showing moderate pneumopericardium observable from lower thorax.

As a part of her workup, it was noted that she had elevated liver function tests, which prompted an abdominal ultrasound that revealed a partially thrombosed portal vein. A follow-up CT of the abdomen and pelvis was then completed. During this CT, the base of the heart was shown to have evidence of pneumopericardium, which led to a CT chest confirming the diagnosis of pneumopericardium with evidence of tamponade (Fig. 1). Tamponade was determined to be primarily contributing to the patient’s hypotension and shock.

**Figure 2:**
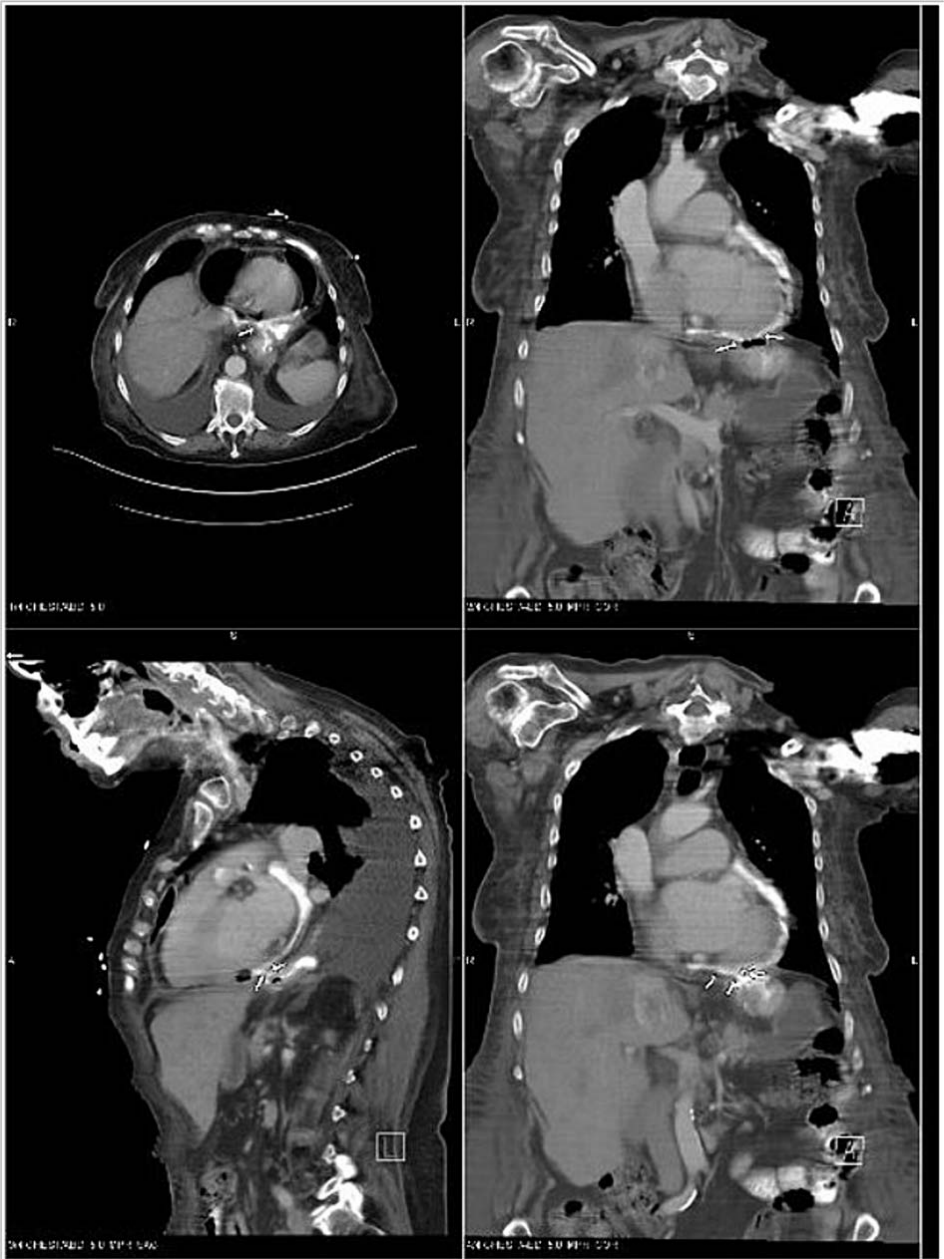
CT Chest with oral contrast demonstrating gastropericardial fistula.

An echocardiogram revealed an EF of 45%–50%. The IVC was dilated and collapsed less than 50% with a sniff, suggesting high right atrial pressure. There was concern that a gastro-pericardial fistula caused the pneumopericardium, so another CT chest with oral contrast was ordered. The CT demonstrated the passage of the oral contrast into the pericardial space, confirming the gastro-pericardial fistula (Fig. 2).

Due to the patient being admitted to a community hospital without cardiothoracic surgery, transfer to a tertiary care center was arranged, and the patient was later transferred. Her clinical course remained tenuous with ongoing hypotension, an ongoing requirement for vasopressors, and worsening hypoxia. She was deemed to be a poor surgical candidate. Her respiratory failure worsened throughout her course, and she eventually passed without being able to receive any surgical intervention.

## Discussion

Gastro-pericardial fistula is a rare condition that can occur as a complication due to Roux-en-Y gastric bypass. The most common risk factor for developing gastro-pericardial fistula is gastroesophageal surgery. Only 65 cases in total have been described in the literature [2]. Additionally, the mortality of this condition is greater than 50% [3, 4]. Case reports remain the most prominent form of documentation due to the condition’s rarity. We performed a literature search of gastro-pericardial fistula case reports over the last 11 years. In the last 11 years, we discovered seven case reports related explicitly to Roux-en-Y procedures [1, 5–8]. Analysis of available information shows that the average time between the Roux-en-Y procedure and complication of pneumopericardium is 9.3 years with a median of 9 years with a range of occurrence between 11 months and 17 years. This is consistent with the previously completed case series from 1988, where the average time until the presentation was seven years [4]. In older case reports, pneumopericardium has been reported up to 27 years after Roux-en-Y [5]. In our patient, the Roux-en-Y procedure occurred 15 years prior to presenting. The average age of patients was 47, with no correlation of complications between genders.

The clinical presentation of a gastro-pericardial fistula can be insidious, as a patient’s initial presentation may have only vague symptoms of weakness and fatigue, dyspepsia, epigastric pain, and clinical findings of iron-deficient microcytic anemia [9]. Further disease advancement can result in bacteremia if the fistula progresses to the myocardium [7]. In our patient, the initial presentation was vague and would not typically lead to the direct diagnosis of gastro-pericardial fistula. No classical signs of tamponade were present on admission. The final diagnosis was incidental as a part of the workup of abnormal liver function tests. The delayed nature of this complication contributes to the difficulty in arriving at the correct diagnosis. Our patient completed an esophagogastroduodenoscopy two months prior to admission, and an anastomotic ulcer was noted around 40 cm with a normal jejunum. This sign could have led to an earlier diagnosis if it had been investigated further and a CT with oral contrast was completed. Generally, upper endoscopy alone will identify less than 50% of fistulas, even when investigated early [4]. The initial discovery of this fistula was further clouded by the patient being asymptomatic at the time of the procedure. The treatment for gastro-pericardial fistula involves immediate surgical management to close the fistula. Broad-spectrum antibiotics are administered to cover for gastrointestinal bacteria that may have colonized the pericardium.

In the management of our patient, antibiotics were rapidly administered, given the concern for septic shock and tamponade physiology on patient presentation. The imaging findings of the gastro-pericardial fistula were incidental as part of the workup of her septic shock with a suspected GI source. Surgical intervention was planned, but due to the nature of her late presentation, clinical status, and ongoing deterioration, she was deemed to be a poor surgical candidate, and surgery was not performed. This patient’s case outlines the later complication of bacterial seeding into the pericardium and subsequent shock with tamponade physiology. Awareness of this possible condition could have prompted further investigation once an ulcer was discovered at 40 cm. To help these cases in the future, further research could be aimed at education on risk factors and the subsequent discovery of gastro-pericardial fistulas before they progress to an unrecoverable state.

## Conclusion

Pneumopericardium is a rare complication of patients with gastric bypass due to gastro-pericardial fistula. Out of the cases published, early recognition resulted in early treatment and better outcomes than late presentation. A thorough history and knowledge of this late complication can lead to an earlier diagnosis and subsequent treatment. From our findings, gastro-pericardial fistula occurred an average of 9 years from the initial Roux-en-Y gastric bypass. Given the nature of late presentation, with an average of 9 years and insidious presentation, the best incite to this pathology is a thorough history. This case highlights this rare complication and emphasizes the importance of surgical history and the inclusion of gastro-pericardial fistula in the differential diagnosis of pneumopericardium.
